# MRgHIFU – experimental perivascular volumetric ablation in the liver

**DOI:** 10.1186/2050-5736-3-S1-O83

**Published:** 2015-06-30

**Authors:** Ulrik Carling, Leonid Barkhatov, Frederic Courivaud, Tryggve Storås, Richard Doughty, Eric Dorenberg, Per Kristian Hol, Bjørn Edwin

**Affiliations:** 1 Oslo University Hospital, Oslo, Norway

## Background/introduction

Thermal ablation techniques using heat conduction (e.g. radiofrequency ablation) are sensitive to the cooling effect of blood flow, the heat sink effect. Thermal ablation by high intensity ultrasound guided by magnetic resonance imaging (MRgHIFU), is less dependent on heat conduction, and produces sharply delineated ablated volumes. The aim of this study was to ablate adjacent to large hepatic and portal veins, to study the heat sink effect on the ablation cells, as well as vessel wall patency. A secondary aim was to study features of second sonication cycles.

## Methods

This acute animal study using Norwegian land swine was approved by The Norwegian Animal Research Authority. The pigs were under total intravenous anaesthesia, including muscle relaxation for optimal breath control. MR compatible equipment included tracheostomy, ECG, gastric tube, arterial and venous accesses, urine catheter, and an oesophageal temperature monitor. After pre-procedural preparations, the pigs were positioned in the prone position on the MR table. T1w sequences were performed, and hepatic and portal veins measuring > 5 mm within the reach of the ultrasound waves were identified. Two clusters of 6-7 ablation cells of 8x8x20 mm, were placed around separate vessels in normal liver parenchyma. In one pig, 4x4x10 mm cells were used. Volumetric ablations were performed during one minute breath-hold in exhale, and the cells were ablated twice. A proton frequency shift sequence was performed for temperature monitoring, and three coronal and one sagittal temperature maps were produced by the HIFU system. Based on these maps, the system registered estimated ablation volume and treatment offset (difference in mm between planned and registered location). Expected volume in an 8 mm cell is 0.74 ml. Further, time to temperature peak was assessed on the temperature maps.

**Figure 1:**
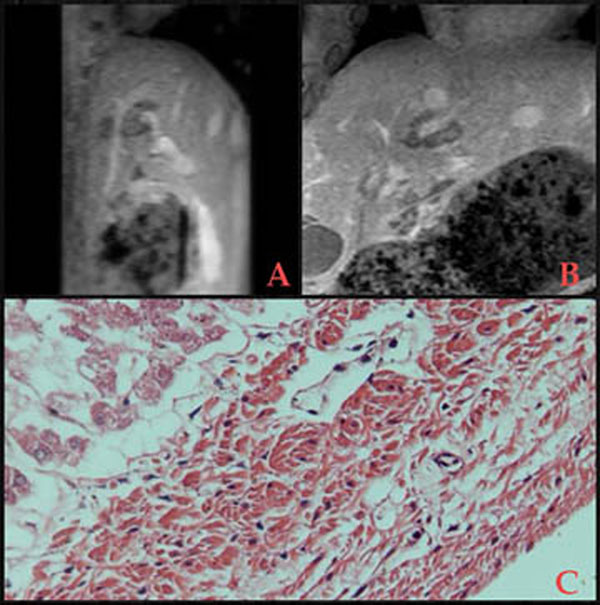
Post-sonication imaging, and histopathology at 400x enlargement (A) Sagittal T1w; ablation cluster adjacent to liver vein (B) Coronal T1w; ablation cluster around portal vein (C) Ablated hepatocytes upper left part, and patent vessel wall in centre.

Postsonication imaging (Figure 1) was performed including diffusion weighted imaging (DWI), and T1w contrast enhanced imaging. The livers were then extracted and put in formalin for histopathological analyses (Figure 1).

## Results and conclusions

A total of 154 ablations, in six pigs, were performed. Of these, 126 ablations were in 8 mm cells, and 28 were in 4 mm cells. In the 126 ablations the median registered ablated volume was 0,3 ml (range 0-2,5), and in 28/154 (25%) no significant volume was registered. Median registered heating offset was 4.2 mm (range 0,3-14,5). Second sonication cycles in 46 cells with a registered ablation volume, had slightly faster heating on central coronal temperature map (0.14ºC/s, p= 0.005). Preliminary histopathology results indicate that liver parenchyma adjacent to vein walls can be ablated, while keeping the vessel wall patent. Further analyses of the sonication data, and the histopathological samples are in progress.

